# Association of rs2910164 Polymorphism in miRNA-146 and rs3746444 Polymorphism in miRNA-499 with Inflammatory Arthritis: A Meta-Analysis

**DOI:** 10.1155/2019/7305750

**Published:** 2019-05-16

**Authors:** Dingjian Wang, Guixia Pan

**Affiliations:** Department of Epidemiology and Biostatistics, School of Public Health, Anhui Medical University, 81 Meishan Road, Hefei, Anhui 230032, China

## Abstract

**Objectives:**

The purpose of this study was to explore the association of miRNA-146 and miRNA-499 polymorphisms with inflammatory arthritis.

**Methods:**

A systematic search of studies on the association of miRNA-146 and miRNA-499 polymorphisms with inflammatory arthritis susceptibility was conducted in PubMed, Web of science, Elsevier ScienceDirect, and Cochrane Library. Eventually, 18 published studies were included. The strength of association between miRNA-146/499 polymorphisms and inflammatory arthritis susceptibility was assessed by odds ratios (ORs) with its 95% confidence intervals (CIs).

**Results:**

A total of 18 case-control studies, consisting of 3385 inflammatory arthritis patients and 4584 controls, were included in the meta-analysis. This meta-analysis showed significant association between miRNA-499 rs3746444 polymorphism and inflammatory arthritis susceptibility in overall population (C vs T, OR: 1.422, 95% CI= 1.159-1.745,* P*=0.001). Similar results were found in subgroup analysis by region. But we did not find association between miRNA-146 rs2910164 polymorphism and inflammatory arthritis susceptibility in overall population (C vs T, OR: 1.061, 95% CI= 0.933-1.207,* P*=0.365).

**Conclusions:**

The present study indicates that miRNA-499 rs3746444 polymorphism is associated with inflammatory arthritis susceptibility. However, there is lack of association between miRNA-146 rs2910164 polymorphism and inflammatory arthritis susceptibility. But, we also find miRNA-146 rs2910164 and miRNA-499 rs3746444 polymorphism are associated with inflammatory arthritis in Middle East. Therefore, more large-scale studies are warranted to replicate our findings.

## 1. Introduction

Inflammatory arthritis is a group of complex diseases, including rheumatoid arthritis (RA), psoriatic arthritis (PsA), juvenile idiopathic arthritis (JIA), etc. And it is characterized by inflammatory cell infiltration into the joints [1, 2]. There is no doubt that environmental factors and genetic factors play an important role in the development of inflammatory arthritis.

MicroRNAs (miRNAs) are small noncoding RNAs, which are about 22 nucleotides long. They play an important role in regulating transcription and translation [3–6]. Some studies indicated that they also participate in cell proliferation, differentiation, and apoptosis [7, 8]. And, miRNAs can cause the degradation or translation repression of their target miRNA by binding to the 3′-untranslated region of specific mRNAs (messenger RNAs) [9–12]. Recently, some studies reported that miRNA may associate with inflammatory arthritis, such as miRNA-146 rs2910164 and miRNA-499 rs3746444. Therefore, we chose miRNA-146 rs2910164 and miRNA-499 rs3746444 to explore the association between its polymorphisms and inflammatory arthritis.

MiRNA-146 is located on human chromosome 5q34 and related to immune regulation, inflammatory signaling pathway, because it can regulate the expression of IL-1 receptor-associated kinase (IRAK1) and IRAK2 and targets TNF receptor-associated factor 6 (TRAF6) [13]. And, miRNA-499 is related to the expression of Interleukin-17 receptor B (IL-17RB), IL-2RB, IL-6, B and T lymphocyte attenuator (BTLA), and peptidyl arginine deiminase 4 (PADI4) [14]. Both miRNA-146 and miRNA-499 are thought to be involved in autoimmune and inflammatory diseases [15].

Considering the results of miRNA-146 and miRNA-499 polymorphisms with inflammatory arthritis are inconsistent [16–20], this discrepancy might be caused by some studies that have small sample size, low statistical power, ethnicity differences, and publication bias. It is necessary to conduct meta-analysis to explore this association.

## 2. Methods

### 2.1. Search Strategy

A systematic search of studies on the association of miRNA-146 and miRNA-499 polymorphisms with inflammatory arthritis susceptibility was conducted in PubMed, Web of science, Elsevier ScienceDirect, and Cochrane Library. Keywords for the search were as follows: (“microRNA” or “miRNA” or “microRNAs”) and (“Inflammatory arthritis”) and (“polymorphism” or “variant” or “genotype” or “SNP” or “mutations”). All relevant studies were retrieved. We screened those studies by reading its titles, abstracts, and contents carefully.

### 2.2. Eligibility Criteria

The inclusion criteria were (i) studies involving the association between miRNA gene polymorphisms and inflammatory arthritis; (ii) case-control study; (iii) studies based on human; (iv) studies providing the detailed relevant genotype data of both case group and control group. Studies were excluded if (i) the study was a review, editorial, abstract, case report, or unpublished article; (ii) studies were nonhuman studies or animal experiments or cell experiments; (iii) studies had no controls or no detailed relevant genotype data.

### 2.3. Data Extraction

The data of the eligible studies were extracted by one investigator (Mr.Wang). The following information were collected: first author's name; year of publication; type of disease; country; region; genotyping methods; number of cases and controls in each study; characteristics of controls; category of polymorphisms and other additional information.

### 2.4. Quality Assessment

The included studies in this meta-analysis were evaluated by another inspector (Pan). The quality assessment was based on the modified Newcastle-Ottawa Quality Assessment Scale (NOS). The scale consists of eight multiple-choice questions that involve subjects' selection, comparability in cases and controls, and assessment of exposure. High-quality response earns a point, totaling up to nine points (the comparability question earns up to two points). The higher score indicates better quality.

### 2.5. Statistical Analysis

All statistical analyses were performed by Stata 12.0 (StataCorp, College Station, TX, USA) software. OR and 95% CI were used to evaluate the strength of association between miRNA-146/499 polymorphisms with inflammatory arthritis susceptibility under different genetic models. The effect of heterogeneity was evaluated using *I*^2^ statistics. Heterogeneity was recognized as statistically significant when *I*^2^ > 50%. According to the value of *I*^2^, we chose fixed-effects or random-effects model. All subgroups were analyzed. Sensitivity analysis was used to evaluate the influence of individual study on the overall OR. Publication bias was assessed by Funnel's plot, Begg's test and Egger's test. An asymmetric plot and the *P* value of Egger's test or Begg's test less than 0.05 were considered as significant publication bias.

## 3. Results

### 3.1. Literature Search

**Figure 1:**
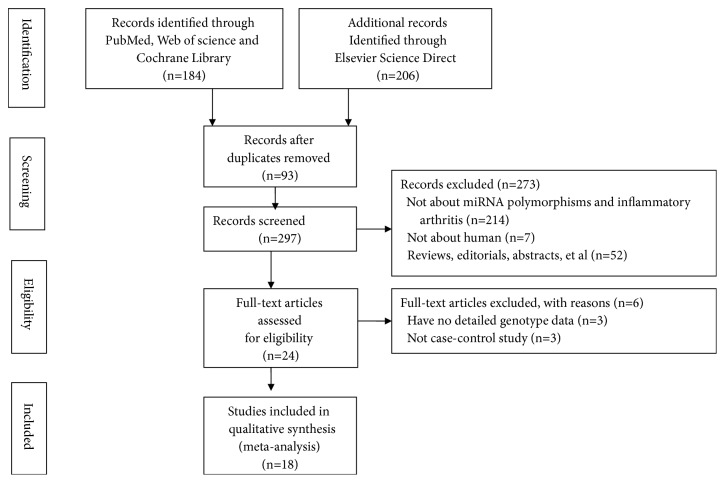
A flowchart of the included and excluded studies.

A total of 390 studies were retrieved from PubMed, Web of science, Elsevier Science Direct and Cochrane Library. Finally, 18 eligible studies were included in this meta-analysis. A flowchart of the included and excluded studies was shown in Figure 1.

### 3.2. Characteristics of the Included Studies

Table 1 showed the main features of those included studies. Those studies were all English articles, which have been published from 2010 to 2018. All studies included in this meta-analysis were case-control studies. Eighteen studies involving 3385 inflammatory arthritis patients and 4584 controls were included in this meta-analysis.

### 3.3. Meta-Analysis of Association between miRNA-146 rs2910164 Polymorphism and Inflammatory Arthritis

**Figure 2:**
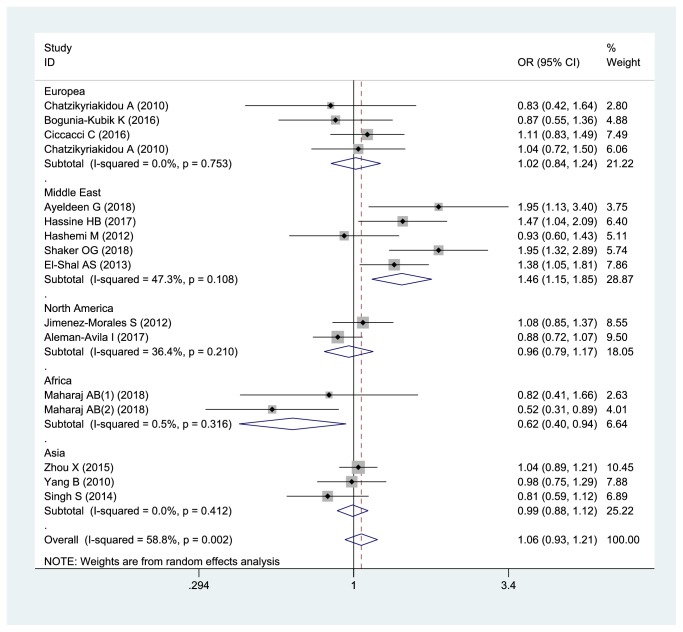
The forest plot about association between miRNA-146 rs2910164 and inflammatory arthritis under allelic model (G vs C).

Meta-analysis indicated that there is lack of association between miRNA-146 rs2910164 polymorphism and inflammatory arthritis susceptibility in the overall population (G vs C, OR: 1.061, 95% CI= 0.933-1.207,* P*=0.365; GC+CC vs GG, OR: 0.919, 95% CI=0.762-1.110,* P*=0.381; CC vs GG+GC, OR: 0.935, 95% CI=0.821-1.065,* P*=0.310). After stratifying by region, it only showed the association between miRNA-146 rs2910164 polymorphism and inflammatory arthritis susceptibility in Middle East (Figure 2). This may be due to the differences in geographic region, ethnicity, and sample size.

### 3.4. Meta-Analysis of Association between miRNA-499 rs3746444 Polymorphism and Inflammatory Arthritis

**Figure 3:**
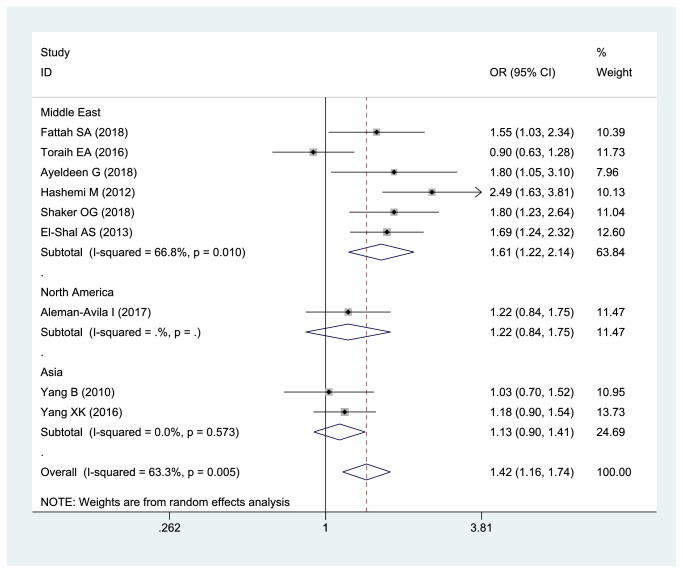
The forest plot about association between miRNA-499 rs3746444 and inflammatory arthritis under allelic model (C vs T).

Meta-analysis indicated significant association between miRNA-499 rs3746444 polymorphism and inflammatory arthritis susceptibility in the overall population (C vs T, OR: 1.422, 95% CI= 1.159-1.745,* P*=0.001; TC +CC vs TT, OR: 1.409, 95% CI=1.072-1.852,* P*=0.014; CC vs TT+TC, OR: 1.872, 95% CI=1.420-2.467,* P*<0.001). After stratifying by region, the association between miRNA-499 rs3746444 polymorphism and inflammatory arthritis susceptibility was still extant in Middle East (Figure 3). But, we did not find this association in Asia and North America.

### 3.5. Heterogeneity and Publication Bias

**Figure 4:**
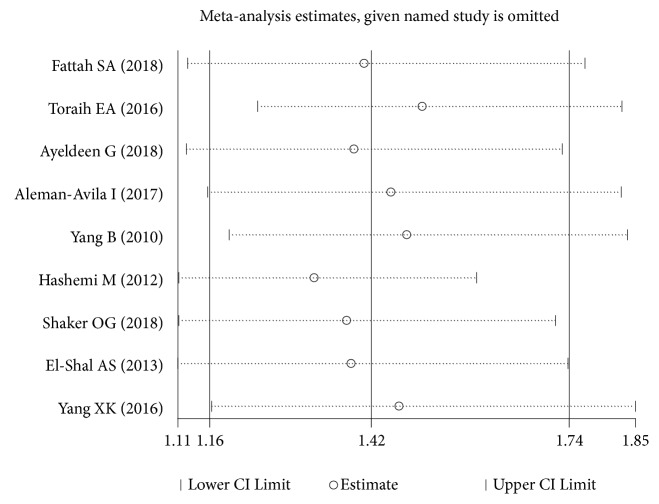
The plot of sensitivity analysis about association between miRNA-499 rs3746444 and inflammatory arthritis.

The results of heterogeneity test were shown in Table 2. And the results of Begg's test and Egger's test were also shown in Table 2. As for some studies that had heterogeneity (*I*^2^ > 50%), the random-effects models were performed; others were analyzed by fixed-effects model. As for miRNA-499 rs3746444 polymorphism, the plot of sensitivity analysis was shown in Figure 4. And there was no significant evidence of publication bias found in the meta-analysis.

## 4. Discussion

Both environmental factors and genetic factors are related to the development of inflammatory arthritis. As for miRNAs, it is reported that they are involved in cancers and autoimmune diseases [15, 21]. But some recent studies about miRNA-146/499 polymorphisms and inflammatory arthritis are inconsistent; it needs us to explore the real association between miRNA-146/499 polymorphisms and inflammatory arthritis by conducting this meta-analysis.

Present meta-analysis included 18 relevant studies, 9 only about miRNA-146 rs2910164, 3 only about miRNA-499 rs3746444, and 6 about both miRNA-146 rs2910164 and miRNA-499 rs3746444. There were 5 studies not met Hardy–Weinberg equilibrium; we did not exclude those 5 studies. We conducted subgroup analysis according to HWE and found that the results are similar (Table 2).

Of course, there were some limitations in this meta-analysis. First, we searched literature based on English; it may cause language bias. Second, the number of included studies was not sufficient, which could not conduct comprehensive analysis. Third, our data of meta-analysis were from retrospective research, which may be related to the methodological deficiencies. Finally, our meta-analysis did not take the interactions between environmental factors and genetic factors into account. Therefore, the results should be interpreted with caution.

In conclusion, the present study indicates that miRNA-499 rs3746444 polymorphism is associated with inflammatory arthritis susceptibility. However, there is lack of association between miRNA-146 rs2910164 polymorphism and inflammatory arthritis susceptibility. But, we find miRNA-146 rs2910164 and miRNA-499 rs3746444 polymorphism are associated with inflammatory arthritis in Middle East. Therefore, it is necessary to conduct more large-sample and more high-quality studies to further validate our findings.

## Figures and Tables

**Table 1:** The main features of included studies.

Author	Year	Disease	Country	Region	Genotyping methods	Number of Cases	Number of controls	Characteristics of controls	Polymorphisms	NOS score
Chatzikyriakidou A	2010	RA	Greece	Europe	PCR–RFLP	136	147	Healthy	rs2910164(miRNA-146)	6

Yang XK	2016	RA	China	Asia	TaqMan	386	576	Healthy	rs3746444(miRNA-499)	7

Singh S	2014	JIA-ERA	India	Asia	PCR–RFLP	150	216	Healthy	rs2910164(miRNA-146)	7

El-Shal AS	2013	RA	Egypt	Middle East	PCR–RFLP	217	245	Healthy	rs2910164(miRNA-146), rs3746444(miRNA-499)	6

Shaker OG	2018	RA	Egypt	Middle East	TaqMan	104	112	Healthy	rs2910164(miRNA-146), rs3746444(miRNA-499)	7

Hashemi M	2012	RA	Iran	Middle East	T-ARMS-PCR	104	110	Healthy	rs2910164(miRNA-146), rs3746444(miRNA-499)	6

Yang B	2010	RA	China	Asia	PCR–RFLP	208	240	Healthy	rs2910164(miRNA-146), rs3746444(miRNA-499)	7

Aleman-Avila I	2017	RA	Mexico	North America	TaqMan	412	486	Healthy	rs2910164(miRNA-146), rs3746444(miRNA-499), rs11614913(miRNA-196)	7

Zhou X	2015	RA	China	Asia	NA	598	821	NA	rs2910164(miRNA-146)	5

Hassine HB	2017	RA	Tunisia	Middle East	PCR–RFLP	165	150	Healthy	rs2910164(miRNA-146)	6

Maharaj AB(1)	2018	PsA	South Africa	Africa	PCR–RFLP	84	62	Healthy	rs2910164(miRNA-146)	6

Maharaj AB(2)	2018	PsA	South Africa	Africa	PCR–RFLP	32	38	Healthy	rs2910164(miRNA-146)	6

Jimenez-Morales S	2012	JRA	Mexico	North America	TaqMan	210	531	Healthy	rs2910164(miRNA-146)	7

Ciccacci C	2016	RA	Italy	Europe	TaqMan	192	298	Healthy	rs2910164(miRNA-146)	7

Ayeldeen G	2018	RA	Egypt	Middle East	real-time PCR	52	56	Healthy	rs2910164(miRNA-146), rs3746444(miRNA-499)	6

Toraih EA	2016	RA	Egypt	Middle East	TaqMan	95	200	Healthy	rs3746444(miRNA-499), rs11614913(miRNA-196)	6

Fattah SA	2018	RA	Egypt	Middle East	PCR–RFLP	100	100	Healthy	rs3746444(miRNA-499)	6

Bogunia-Kubik K	2016	RA	Poland	Europe	PCR–RFLP	111	130	Healthy	rs2910164(miRNA-146)	6

Chatzikyriakidou A	2010	PsA	Greece	Europe	PCR–RFLP	29	66	NA	rs2910164(miRNA-146)	6

HWE: Hardy–Weinberg equilibrium; NOS: Newcastle-Ottawa Quality Assessment Scale.

**Table 2:** The results of meta-analysis, heterogeneity test, and publication bias.

polymorphism	Number of studies	Comparable genotype	No. of Cases/Controls	Test of association			Test of heterogeneity		Test of publication bias				Model
									Begg's test		Egger's test		
				OR (95%CI)		*P*	*I* ^2^	*P*_H_	Z	*P*	t	*P*	
rs2910164 (miRNA-146)													
Overall	16	G vs C	2802/3708	1.061(0.933-1.207)		0.365	58.80%	0.002	0.14	0.893	0.60	0.559	R
Region													
Europe	4	G vs C	468/641	1.017(0..836-1.236)		0.868	0.00%	0.753	1.02	0.308	-2.93	0.100	F
Asia	3	G vs C	956/1277	0.991(0.877-1.120)		0.890	0.00%	0.412	1.04	0.296	-1.89	0.309	F
Middle East	5	G vs C	642/673	1.449(1.229-1.709)		0.001	47.30%	0.108	0.24	0.806	0.53	0.630	F
Africa	2	G vs C	116/100	0.615(0.405-0.935)		0.023	0.50%	0.316	0.00	1.000	NA	NA	F
North America	2	G vs C	620/1017	0.955(0.820-1.111)		0.551	36.40%	0.210	0.00	1.000	NA	NA	F
HWE													
Y	13	G vs C	2524/3359	1.053(0.916-1.211)		0.469	61.90%	0.002	0.18	0.855	0.99	0.344	R
Overall	16	GC+CC vs GG	2802/3708	0.919(0.762-1.110)		0.381	57.20%	0.002	-0.05	1.000	0.05	0.963	R
Region													
Europe	4	GC+CC vs GG	468/641	1.000(0.784-1.276)		0.998	0.00%	0.606	1.70	0.089	13.66	0.005	F
Asia	3	GC+CC vs GG	956/1277	0.980(0.795-1.209)		0.851	0.00%	0.869	0.00	1.000	0.16	0.896	F
Middle East	5	GC+CC vs GG	642/673	0.530(0.320-0.877)		0.014	64.30%	0.024	0.73	0.462	-0.99	0.394	R
Africa	2	GC+CC vs GG	116/100	1.915(1.107-3.314)		0.020	49.40%	0.160	0.00	1.000	NA	NA	F
North America	2	GC+CC vs GG	620/1017	1.009(0.735-1.385)		0.956	57.20%	0.127	0.00	1.000	NA	NA	R
HWE													
Y	13	GC+CC vs GG	2524/3359	0.942(0.780-1.137)		0.532	55.80%	0.007	0.06	0.951	0.02	0.982	R
Overall	16	CC vs GG+GC	2802/3708	0.935(0.821-1.065)		0.310	34.60%	0.086	1.22	0.224	0.98	0.343	F
Region													
Europe	4	CC vs GG+GC	468/641	0.914(0.581-1.439)		0.699	0.00%	0.839	-0.34	1.000	0.23	0.837	F
Asia	3	CC vs GG+GC	956/1277	1.149(0.803-1.645)		0.447	59.10%	0.087	1.04	0.296	2.69	0.227	R
Middle East	5	CC vs GG+GC	642/673	0.631(0.483-0.823)		0.001	0.00%	0.431	0.24	0.806	-0.60	0.590	F
Africa	2	CC vs GG+GC	116/100	2.211(0.674-7.256)		0.191	0.00%	0.581	0.00	1.000	NA	NA	F
North America	2	CC vs GG+GC	620/1017	1.151(0.837-1.582)		0.387	0.00%	0.739	0.00	1.000	NA	NA	F
HWE													
Y	13	CC vs GG+GC	2524/3359	0.959(0.834-1.103)		0.556	40.20%	0.066	0.55	0.583	0.37	0.722	F
rs3746444 (miRNA-499)													
Overall	9	C vs T	1678/2125	1.422(1.159-1.745)		0.001	63.30%	0.005	1.15	0.251	1.30	0.234	R
Region													
Asia	2	C vs T	594/816	1.128(0.904-1.406)		0.286	0.00%	0.573	0.00	1.000	NA	NA	F
Middle East	6	C vs T	672/823	1.614(1.219-2.137)		0.001	66.80%	0.010	0.75	0.452	0.77	0.485	R
North America	1	C vs T	412/486	1.215(0.845-1.748)		0.294	NA	NA	NA	NA	NA	NA	F
HWE													
Y	7	C vs T	1479/1815	1.390(1.214-1.591)		0.001	31.00%	0.191	0.30	0.764	0.99	0.368	F
Overall	9	TC+CC vs TT	1678/2125	1.409(1.072-1.852)		0.014	67.50%	0.002	0.10	0.917	1.03	0.335	R
Region													
Asia	2	TC+CC vs TT	594/816	1.138(0.891-1.454)		0.301	0.00%	0.374	0.00	1.000	NA	NA	F
Middle East	6	TC+CC vs TT	672/823	1.634(1.062-2.513)		0.026	73.40%	0.002	0.75	0.452	0.10	0.924	R
North America	1	TC+CC vs TT	412/486	1.188(0.809-1.742)		0.379	NA	NA	NA	NA	NA	NA	F
HWE													
Y	7	TC+CC vs TT	1479/1815	1.399(1.190-1.645)		0.001	38.20%	0.138	0.00	1.000	1.11	0.319	F
Overall	9	CC vs TT+TC	1678/2125	1.872(1.420-2.467)		0.001	0.00%	0.588	0.10	0.917	0.42	0.686	F
Region													
Asia	2	CC vs TT+TC	594/816	1.224(0.551-2.717)		0.619	0.00%	0.495	0.00	1.000	NA	NA	F
Middle East	6	CC vs TT+TC	672/823	1.960(1.455-2.641)		0.001	0.00%	0.462	0.38	0.707	0.66	0.545	F
North America	1	CC vs TT+TC	412/486	3.557(0.369-34.331)		0.273	NA	NA	NA	NA	NA	NA	F
HWE													
Y	7	CC vs TT+TC	1479/1815	1.910(1.334-2.736)		0.001	0.00%	0.696	0.00	1.000	-0.21	0.840	F

R: random-effects model; F: fixed-effects model; Y: yes (studies that suitable for HWE); *P*_H_: the value of *P* in the heterogeneity test.
